# Establishment of a Landscape of UPL5-Ubiquitinated on Multiple Subcellular Components of Leaf Senescence Cell in *Arabidopsis*

**DOI:** 10.3390/ijms23105754

**Published:** 2022-05-20

**Authors:** Wei Lan, Shuai Zheng, Ping Yang, Yuhao Qiu, Yun Xu, Ying Miao

**Affiliations:** Fujian Provincial Key Laboratory of Plant Functional Biology, Fujian Agriculture and Forestry University, Fuzhou 350002, China; 1150538001@fafu.edu.cn (W.L.); 1190514085@fafu.edu.cn (S.Z.); 1190514070@fafu.edu.cn (P.Y.); qiuyuhao@fafu.edu.cn (Y.Q.); 1190514066@fafu.edu.cn (Y.X.)

**Keywords:** integrative analysis, UPL5, subcellular localization, ubiquitination, leaf senescence

## Abstract

Catabolism of macromolecules is a major event in senescent cells, especially involving proteolysis of organelles and abnormally aggregated proteins, circulation of nutrients, and precise control of intracellular environmental balance. Proteasomes are distributed in the nucleus and cytoplasm; however, proteasomes in organelles are limited. In this study, multi-omics proteomic analyses of ubiquitinated proteins enriched by using antibody against “di-Gly-Lys” via a free labeling were used to investigate the global changes of protein levels and ubiquitination modification levels of *upl5* mutant relative to wild-type plant; subcellular localization analysis of UPL5 was found to be located in the nucleus, cytoplasm, and plastid within the cell; and the direct lysine site patterns of UPL5 were screened by the H89R substitution in the tagged ubiquitinated assay. It suggests that UPL5 acting as a candidate of organelle E3 ligase either in the nucleus or cytoplasm or plastid modifies numerous targets related to nuclear transcription and plastid photosynthesis involving in Ca^2+^ and hormone signaling pathway in plant senescence and in response to (a)biotic stress protection.

## 1. Introduction

Cell senescence, including developmental senescence and stress-induced senescence, is triggered by internal and external factors and often involves degradation and remobilization of cellular components. During plant senescence, a visible change of leaf yellowing is an indication of chloroplast damage and chlorophyll degradation. At the molecular level, catabolism of macromolecules is a major event in senescent cells, especially proteolysis. The main proteolysis pathways during plant aging are proteasomes UPS and autophagy, which together promote the turnover of organelles and abnormally aggregated proteins, the circulation of nutrients, and the precise control of intracellular environmental balance [1]. Protein degradation is catalyzed by proteases and proteasomes. Proteases are mainly distributed in multiple-cell regions such as vacuoles, chloroplasts, mitochondria, and the secretory pathway, while proteasomes are distributed in the nucleus and cytoplasm [2]. In addition, proteasome-mediated protein degradation is prevalent throughout the leaf life cycle. Genes encoding proteasome-related subunits are expressed relatively stable during leaf development [3], suggesting that the ability to degrade proteins through proteasomes is existed during cell aging.

Up to now, it has been reported that numerous senescence-associated transcription factors are controlled by 26S proteasome-mediated protein degradation. For example, ORE9 (also known as more axillary growth locus 2, MAX2) is an F-box E3 that targets brassinosteroid-related transcription factors BZR1 and BZR2/BES1 and prompts them to be degraded by 26S proteasomes in *Arabidopsis* [4]. Loss of ORE9 inhibits the expression of a large number of NAC transcription factors, including ATAF1, ANAC019, ANAC055, ANAC072, and others, exhibiting a delay aging phenotype, while the *bes1* mutant exhibits progeria [4,5,6]. Another aging-related RING E3, RING-H2 FINGER A2A, interacts with ANAC019 and ANAC055, thereby limiting their protein levels during plant aging [7]. In addition, the N-end rule pathway of E3 PRT6 (PROTEOLYSIS 6) negatively regulates the onset of leaf senescence and plays an important role in the aging process [8].

*Arabidopsis* HECT-type ubiquitin ligase 5 (UPL5) is a member of the plant HECT-type ubiquitinated E3 ligase family, which is highly expressed in senescent leaves [9]. In comparing the amino acid sequences of animal and plant HECT-type ubiquitin ligases, it is found that there is no member corresponding to UPL5 of *Arabidopsis* HECT-type ubiquitinated E3 ligase family in yeast and animal genomes [10,11]. The plant UPL5 belongs to a clad of itself. Studies have shown that UPL5 can ubiquitinate WRKY53, a key regulatory transcription factor of plant aging and immunity, and promote its degradation by 26S proteasome, thereby inhibiting the process of leaf senescence [12,13,14]. UPL5 is also involved in regulating pathogenic responses [15]; however, its action mechanism is still limited. 

In this study, to in-depth and globally analyze UPL5 function, multi-omics proteomic analyses of ubiquitinated proteins enriched by using antibody against “di-Gly-Lys” via a free labeling was used to investigate the global changes of protein levels and ubiquitination modification levels of *upl5* mutant relative to wild-type (WT) plant; subcellular localization of UPL5 protein was found to be located in multiple regions within the cell; and the direct lysine site patterns of UPL5 were screened by the H89R substitution in the tagged ubiquitinated assay. It indicates that UPL5 acting as a normal E3 ligase modifies numerous targets related to nuclear transcription and plastid photosynthesis involving in Ca^2+^ and hormone signaling pathway in plant senescence and in response to (a)biotic stress protection.

## 2. Results

### 2.1. Loss of UPL5 Declines Globally Ubiquitination of Lysine Sites at Onset of Leaf Senescence

**Figure 1:**
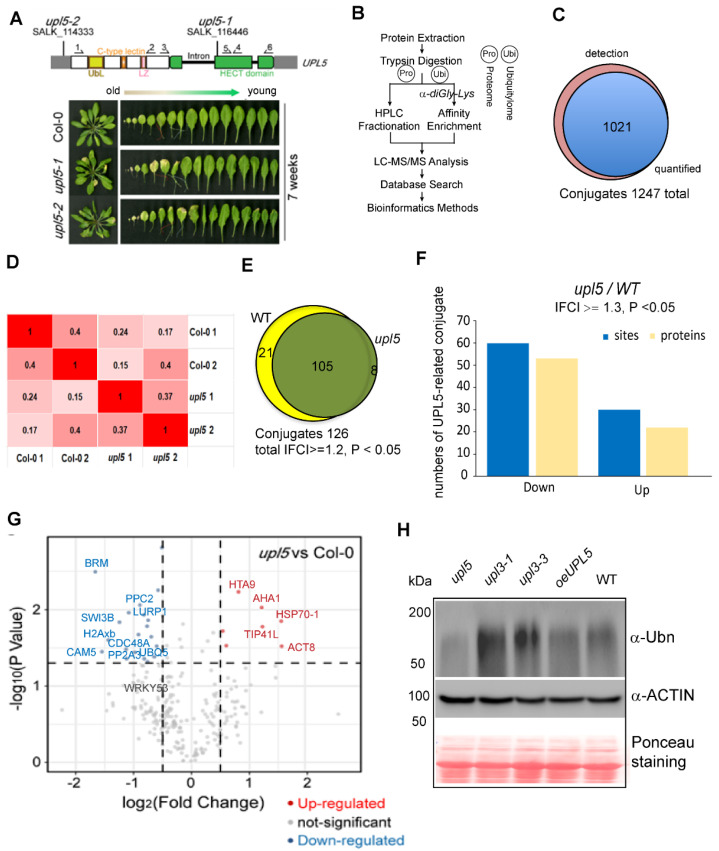
Identification of ubiquitin conjugates in 6-week-old *upl5* plants compared to the wild-type (WT). (**A**) Seven-week-old rosette leaves of *UPL5* gene T-DNA insertion knockout lines. (**B**) A label-free mass spectrometry-based analysis procedure of protein ubiquitination using K-epsilon-GG remnant antibody enrichment approach. (**C**) Venn diagrams showing the enrichment of differentially ubiquitinated proteins in the *upl5* background. (**D**) Pearson coefficient analysis of two replicate samples of the *upl5* and the wild-type. (**E**) Venn diagrams showing the enrichment of differentially ubiquitinated proteins (lCFl ≥ 1.2, *p* value < 0.05) in the *upl5* relative to the WT. (**F**) Distribution of enriched proteins and sites within differential fold-change levels (lCFl ≥ 1.3, *p* value < 0.05) by the *upl5* relative to the WT. (**G**) Volcano plot of individual ubiquitination site showing their *p* value and the log_2_ FC. Dark-grey points are conjugates considered to be “abundant” by their detection in two biological replicates and three technique repeats in either background of the *upl5* and/or the WT. Proteins with a significant decrease or increase in ubiquitination in the *upl5* compared with the WT (*p* value < 0.05) are highlighted in red and blue, respectively. Ubiquitinated targets identified in all WT biological replicates and never or only once in *upl5* mutants, but that were above the significance threshold of *p* value > 0.05, are in grey. The dashed line represents the theoretical situation, where conjugate abundance in the WT and the *upl5* is equal. The horizontal dashed line highlights a *p* value = 0.05. The vertical dashed lines highlight a 1.2-fold (log_2_ FC = 0.26) increase or decrease. (**H**) Immunodetection of global ubiquitinated proteins in WT plants and loss of or gain of *UPL5* mutants using an antibody against polyubiquitin, *upl3* mutants were used as control. Western blotting of ACTIN and Ponceau staining are used as protein loading control.

It has been reported that loss of UPL5 accelerated leaf senescence. *UPL5* gene highly expressed at 6-week-old *Arabidopsis* plants [13]. In order to globally address UPL5 function and identify its substrates, we performed a label-free mass spectrometry (MS)-based analysis of protein ubiquitination using di-Gly-Lys remnant antibody enrichment approach with 6-week-old *upl5* and WT plants (Figure 1A,B). Identified proteins based on their tandem mass spectra matching against the UniProt *Arabidopsis thaliana* Columbia database (current total of 39,211 reads) using the MaxQuant software were listed in Supplementary Dataset S1. Label-free quantification (LFQ), with a false discovery rate (FDR) adjusted to <0.01 and a minimum score for modified peptides set as >40, resulted in a set of over 1247 sites (723 proteins) potential ubiquitinated targets. This was further refined to a subset of 1021 sites (610 proteins) targets by at least two post-translational modifications (PTMs) (Figure 1C; Supplementary Dataset S1).

To identify the alteration of ubiquitin conjugates associated with the *UPL5* mutation, a fold-change greater than 1.2 or less than 1/1.2 and *p* value < 0.05 of two replicates was used to filter conjugate targets in the library, whose ubiquitination was up-regulated or down-regulated (Figure 1D). All the differentially ubiquitinated conjugates (DUCs) 126 sites (105 proteins) in the *upl5* relative to the WT (|FC| ≥ 1.2, *p* value < 0.05) were shown in Figure 1E and Supplementary Dataset S2. Among them, ubiquitination of 88 sites (72 proteins) was found to be down-regulated, and ubiquitination of 38 sites (33 proteins) was up-regulated in the *upl5*, compared to the WT (Figure 1F). This result was verified by global ubiquitination immunodetection using an anti-polyubiquitin antibody, in which deletion of UPL5 led to declined signals of global protein ubiquitination levels, whereas overexpressing *UPL5* increased poly-ubiquitin signals (Figure 1G). Fold-change levels relative to the statistic *p* value of individual ubiquitinated site were plotted to give more details on the dataset (Figure 1G). Of them was the fact that ubiquitin-conjugating enzyme 5 (UBQ5) and WRKY53, a known target of UPL5 [13], appeared in the dataset of ubiquitome with down-regulated ubiquitination level, indicating that the quality of ubiquitomic datasets was properly sound, although a high 0.2–0.4 variant was shown between two replicates. Together, this result means that UPL5 is a typical ubiquitin E3 ligase and has a function of polyubiquitination in ubiquitin proteasome 26S pathway.

### 2.2. UPL5 Affects DUCs Enriched in Endoplasmic Reticulum (ER) Associated Degradation and Calcium Signal Pathway 

**Figure 2:**
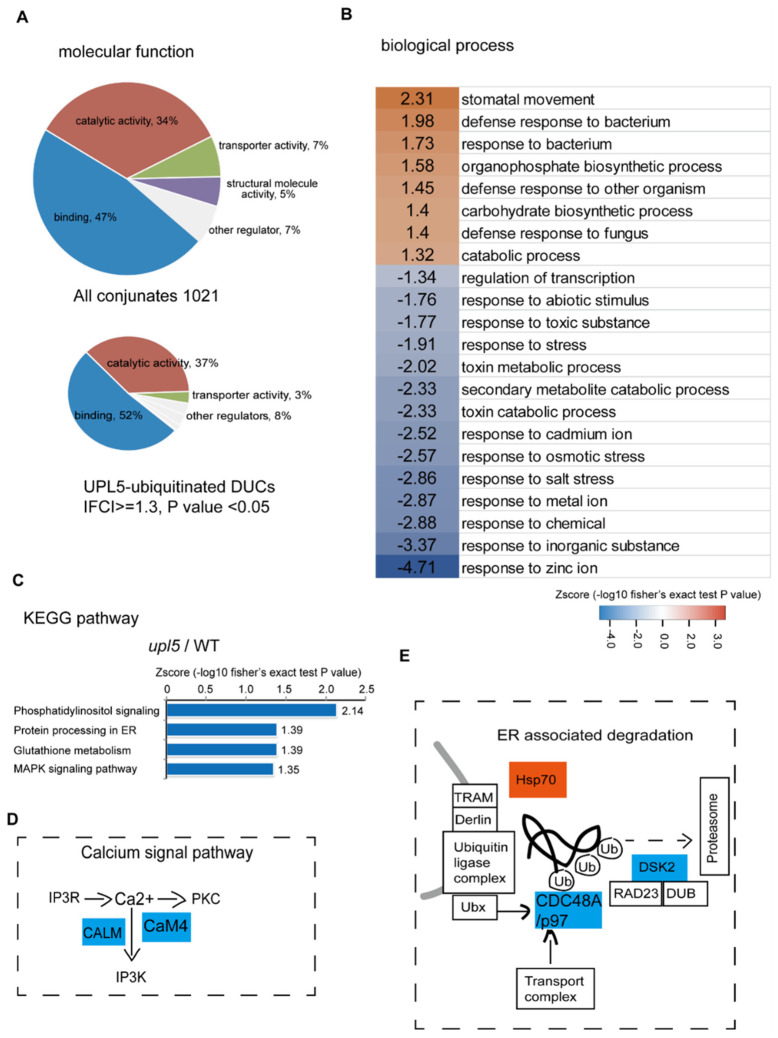
Gene Ontology (GO) term and KEGG pathway enrichments for the UPL5-associated ubiquitin conjugates (changed fold 1.2, *p* value < 0.05). (**A**) GO term enrichment in categories of molecular functions of UPL5-associated ubiquitin conjugates. (**B**) GO term enrichment in categories of biological process of UPL5-associated ubiquitin conjugates. (**C**) KEGG pathway of UPL5-associated ubiquitin conjugates differentially ubiquitinated conjugates (DUCs). (**D**) The UPL5-regulated ubiquitin conjugates of the *upl5*/WT mapped in calcium signal pathway. (**E**) The UPL5-regulated ubiquitin conjugates of the *upl5*/WT mapped in endoplasmic reticulum (ER) associated degradation pathway.

Gene Ontology (GO) term enrichment of all conjugates (Supplementary Dataset S1) showed that the molecular functions of assembled ubiquitin conjugates were related to protein-protein interactions (47%), catalytic activity (34%), transport activity (7%), structural molecule activity (5%), and others (7%). However, for the DUCs (|FC| ≥ 1.3, *p* value < 0.05; Supplementary Dataset S2) of the *upl5* relative to the WT, the function enrichments of protein-protein interaction and catalytic activity were increased to 52% and 37%, respectively (Figure 2A), implying a major involvement in molecular interaction for the UPL5-regulated targets. Further assignment to biological processes showed that the DUCs include mainly proteins involved in regulation of transcription, small molecule metabolism and response to stresses (Figure 2B). Hence, it is plausible that UPL5 maintains the ubiquitination status of proteins related to regulatory factors and stresses.

This notion was further validated via KEGG pathway analysis (Figure 2C). The most significantly enriched pathways of UPL5-ubiquitinated DUCs were phosphatidylinositol (IP3K) signaling, protein processing in ER, glutathione metabolism, and MAPK signaling pathways. The ubiquitinated proteins of the IP3K pathway, such as calmodulin protein CALM (CAM5) and CaM4, and that of ER-associated degradation pathway, such as an upregulated chaperon heat shock protein Hsp70 and two down-regulated CDC48A (p97 in yeast and human) and DSK2 in *upl5* plants (Figure 2D,E). It suggests that UPL5 is involved in calcium related signaling pathway and ER-associated degradation pathway.

### 2.3. UPL5 Affects DUCs Enriched in Nucleus, Cytoplasm, and Plastids

**Figure 3:**
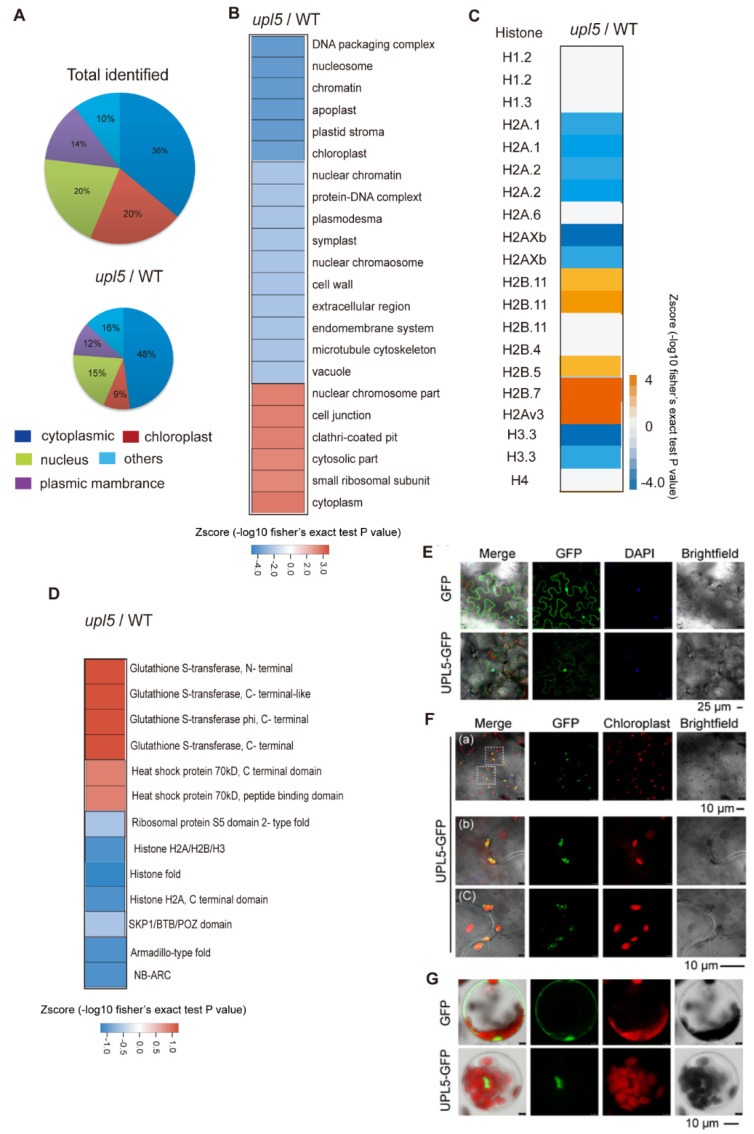
UPL5-related differentially ubiquitinated conjugates (DUCs) associated with cellular components. (**A**) Distribution of UPL5-related DUCs associated with cellular components. (**B**) UPL5-related DUCs enriched in nucleus, plastid, and cytoplasm. (**C**) UPL5-related DUCs enriched in histone variants. (**D**) Gene Ontology terms enrichment analysis of enriched protein domains of DUCs (changed fold 1.2, *p* value < 0.05) via InterProScan. (**E**–**G**) Subcellular localization of UPL5 in nucleus of *Arabidopsis* leaves epidermal cells (**E**) and protoplasts (**G**), as well as in chloroplasts (**F**), transiently expressing a UPL5 C-terminal GFP fusion. The GFP-alone expressing leaves sample is used as control. DAPI staining presents in the nucleus, autofluorescence signal of chlorophyll presents in chloroplast.

Cellular components of our *Arabidopsis* ubiquitomic proteins (Supplementary Dataset S1) can be classified as cytoplasmic (36%), nuclear (21%), chloroplast (19%), membrane (14%), and others (10%). Among these, the percent ratio of the UPL5-related DUCs (|FC| ≥ 1.2, *p* value < 0.05; Supplementary Dataset S2) was increased in cytoplasmic (48%) and plasma membrane (16%) but was slightly declined in nuclear section (19%) and largely decreased in plastid section (9%) (Figure 3A,B). The detail insight of the nuclear section, the UPL5-related DUCs distinctly clustered in histone variants such as hypoubiquitinated H2A.1, H2Axb, H1, H3, and hyperubiquitinated H2A variants, H2B in the *upl5* relative to the WT, (Figure 3C). We further screened protein domains of the DUCs using InterProScan and identified several UPL5-hyperubiquitinated (reduced ubiquitination in the *upl5* background) protein domains, including histone H2A/H2B/H3 domain, histone fold, nucleotide-binding NB-ARC, and armadillo-type fold domain (Figure 3D blue), whilst the UPL5–hypoubiquitinated conjugates (enhanced ubiquitination in the *upl5* background) contained GST domain and HSP70 domain (Figure 3D orange). It suggests that the UPL5-ubiquitinated conjugates were more enriched in the nucleus and cytoplasm.

To clarify subcellular localization of UPL5, UPL5 fusion GFP plasmid was transformed transiently in *Arabidopsis* leaves (epidermal cells and protoplasts) and showed that UPL5 seemed to be localized either in the nucleus (Figure 3E,G), in plastids (Figure 3F), or in cytoplasm [13]. Therefore, UPL5 might have functions both for nuclear proteins (e.g., histone variants, which might be those regulatory functioning in chromatin remodeling, chromosome regulation, and transcriptional complexes) and for cytoplasmic proteins (e.g., ribosomal protein S5, GST domain protein and SKP1/BTB/POZ domain protein, as well as WRKY53 that ubiquitinated by UPL5 in cytoplasm [13]), as well as for plastid proteins (e.g., LHCB protein, PETE1 proteins) (Supplementary Dataset S2).

### 2.4. UPL5 Affects Various Patterns of Lysine Ubiquitination

Ubiquitin can be attached to substrate proteins as a single moiety or polymeric chains and adopt distinct conformations and lead to different functions in cells [16]. In order to gain insight into ubiquitinated lysine site pattern of UPL5, the H89R substitution in the tagged ubiquitinated assay was used here to enable the detection of ubiquitination sites (“footprints”) (Kub) and identify a consensus ubiquitin attachment sequence [17]. By scanning all generated datasets, we identified 2778 ubiquitinated sites in total (Supplementary Dataset S1). Among them, 2359 sites were quantified, 1641 of the 2359 sites were in *upl5* plants, 414 sites were differentially displayed relative to WT plants with a cut-off log_2_FC of 1.5.

**Figure 4:**
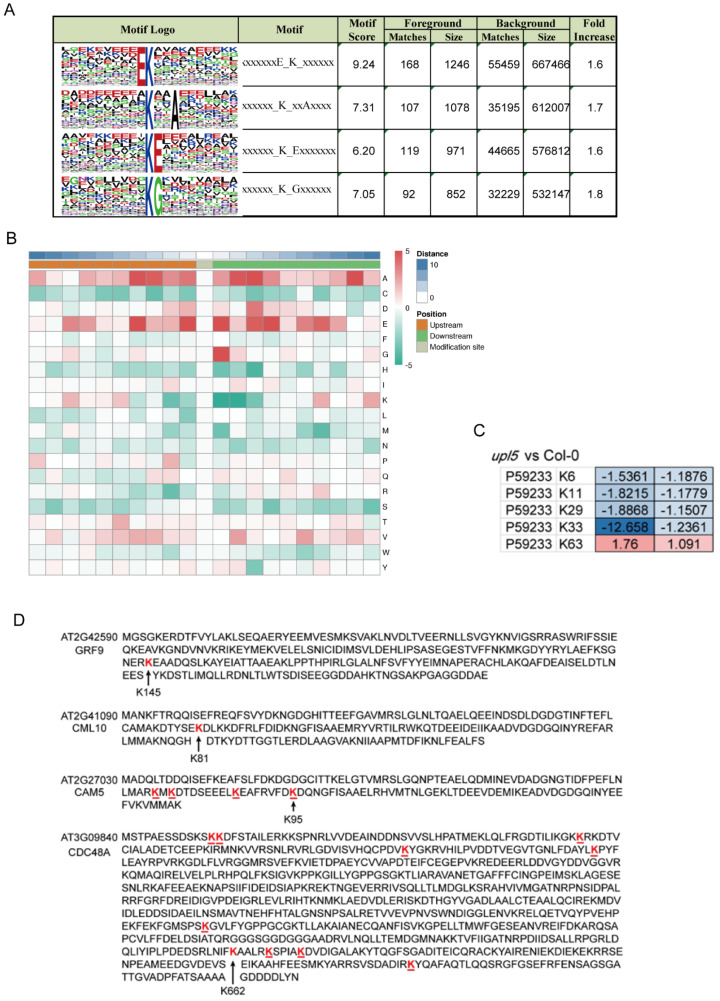
Lysine footprints (Kub-sites) of UPL5-related differentially ubiquitinated conjugates (changed fold 1.5, *p* value < 0.05). (**A**) The consensus ubiquitin attachment motif identified by the MEME Suite (http://meme-suite.org) (accessed on 2 January 2019). (**B**) Heatmap of the enrichment of amino acids upstream and downstream of differentially ubiquitinated conjugates sites (changes fold 1.5, *p* value < 0.05) in the *upl5* relative to the wild-type. Red indicates that the amino acid is significantly enriched near the modification site, and green indicates that the amino acid is significantly reduced near the modification site. (**C**) Ubiquitin-conjugating enzyme 5 lysine footprints of two replicates in the *upl5* relative to the wild-type. (**D**) List of several examples apparently ubiquitin attachment sites and motif in the identified proteins.

We identified 53 ubiquitinated modification sites on 47 differentially ubiquitinated proteins in the *upl5* relative to the WT (log_2_ FC ≥ 1.5, *p* value < 0.05) (Supplementary Dataset S2). Motif analysis around the modified lysine using MEME identified a consensus ubiquitin attachment sequence in 39 of the 53 sites (Figure 4A,B) that strongly matched the c-K-x-E/D/G ubiquitination motif (where c and x represent a hydrophobic and any amino acid, respectively), which was a prevalent motif in yeast and animal ubiquitinated targets [17]. However, the remaining 14 sites (26%) were unrelated to this motif, indicating that non-canonical sites also existed. Interestingly, loss of *UPL5* altered the ubiquitin enrichment level of K6, K11, K29, K33, and K63 sites of UBQ5 protein (Figure 4C). Among them, downregulated K33 and K63 belonged to c-K-x-E/D/G ubiquitination motif, and K6, K11, and K29 belonged to a non-canonical site pattern. The downregulated Kub-sites of DUCs such as GRF9 (AT2G42590) K145 site, CML10 (AT2G41090) K81 site, and CAM5 (AT2G27030) K95 site belonged to c-K-x-E/D/G. In addition, CDC48A had 9 putative Kub-sites, besides, an unidentified site of CDC48A-K662 site was downregulated 1.67–1.95 folds in the dataset (Figure 4D). It suggests that UPL5 could affect various patterns of Kub-sites modification.

### 2.5. UPL5 Affects Diferentially Expressed Proteins (DEPs) Enrichment in Calcium and Hormone Signaling Pathway

**Figure 5:**
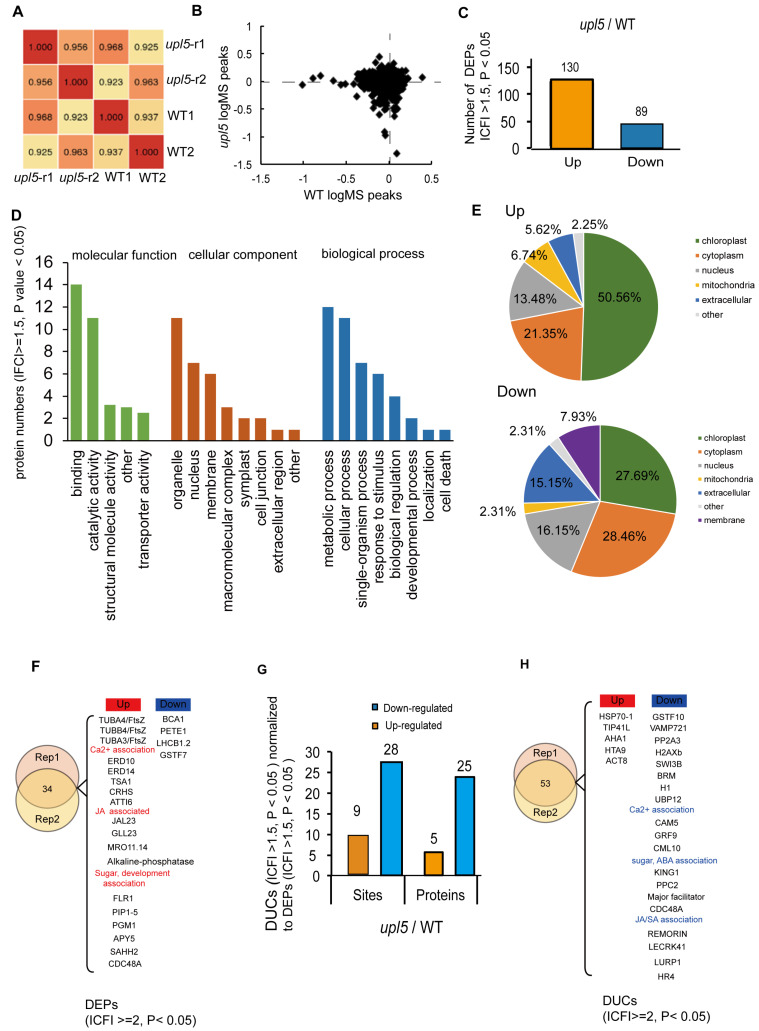
The proteome analysis of the *upl5* relative to the wild-type (WT). (**A**) Pearson coefficient analysis of two replicate samples. (**B**) Enrichment peak of proteins in the *upl5* relative to the WT. (**C**) Numbers of differentially expressed proteins (DEPs) (Fold changed ≥ 1.5, *p* value < 0.05) of the *upl5* relative to the WT (Left). (**D**) GO analysis of DEPs (Fold changed ≥ 1.5, *p* value < 0.05) of the *upl5* relative to the WT. (**E**) Cellular components of DEPs (Fold changed ≥ 1.5, *p* value < 0.05) of the *upl5* relative to the WT. (**F**) The interesting candidates of DEPs (Fold changed ≥ 2, *p* value < 0.05) of the *upl5* relative to the WT. (**G**) Numbers of differentially ubiquitinated conjugates (DUCs) (Fold changed ≥ 1.5, *p* value < 0.05) normalized to DEPs (Fold changed ≥ 1.5, *p* value < 0.05). Orange, upregulated; blue, downregulated. (**H**) The interesting candidates of DUCs/DEPs (Fold changed ≥ 2, *p* value < 0.05) of the *upl5* relative to the WT. Orange, upregulated; blue, downregulated.

To examine whether UPL5 altered the protein level of the substrate candidates of UPL5 in various components of the cell, we compared the total proteome of 6-week-old *upl5* and WT plants (the same as ubiquitomic materials) by tandem MS using the precursor ion intensity of the MS1 scans for quantification. Altogether, 3557 *Arabidopsis* proteins could be reproducibly identified and quantified in both samples by our liquid chromatography-mass spectrometry regime analyzed in duplicate (Figure 5A; Supplementary Dataset S3). Only 33 differentially expressed proteins (DEPs, |FC| ≥ 1.5, *p* value < 0.05) of proteome dataset from the *upl5* relative to the WT (Figure 5B,C; Supplementary Dataset S4) were collected. The GO analysis of the overlapping DEPs (|FC| ≥ 1.5, *p* value < 0.05) of two replicates showed that the molecular functions of them were enriched for protein binding and catalytic activity, and the biological processes of them were enriched in metabolic process, cellular process, single-organism process, response to stimulus, biological regulation, developmental process, localization, and cell death (Figure 5D). Of these, the upregulated DEPs were enriched in microtubule-based process, mitotic cell cycle, regulation of cell death, and cellular response to jasmonic acid (JA) and gravity; and the downregulated DEPs were enriched in the photosynthesis, protein-chromophore linkage, response to redox stress and calcium stimulus, as well as regulation to stomatal movement (the GO analysis independently of two replicates shown in Appendix A and Appendix B). More interestingly, in the cellular component category of GO analysis, numerous upregulated DEPs (|FC| ≥ 1.5, *p* value < 0.05) were increasingly accumulated in chloroplast (50.56%) and mitochondrion (6.74%), the downregulated DEPs were increasingly enriched in cytoplasm (28.46%), the nucleus (15.15%), and extracellular (16.15%), suggesting UPL5 might affect the displacement of proteins or protein trafficking among organelle, nucleus, and cytoplasm (Figure 5E).

**Figure A1:**
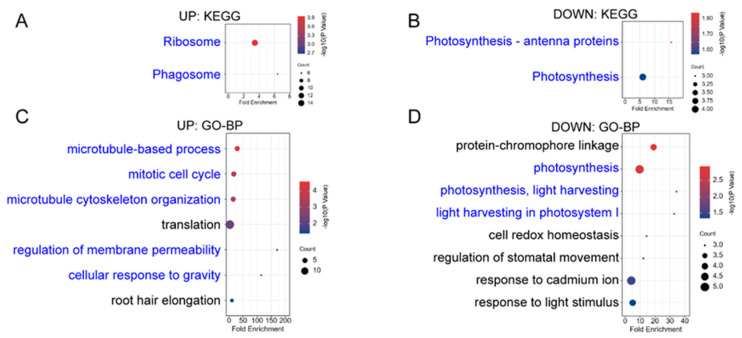
KEGG and GO enrichment analysis of the first repetition of differentially expressed proteins (DEPs-Rep1, ICFI ≥ 1.5) in “*upl5-1* vs. Col-0” group. (**A**,**B**) KEGG enrichment analysis of up-regulated DEPs-Rep1 (**A**) and down-regulated DEPs-Rep1 (**B**); (**C**,**D**) GO enrichment analysis-biological process of up-regulated DEPs-Rep1 (**C**) and down-regulated DEPs-Rep1 (**D**).

**Figure A2:**
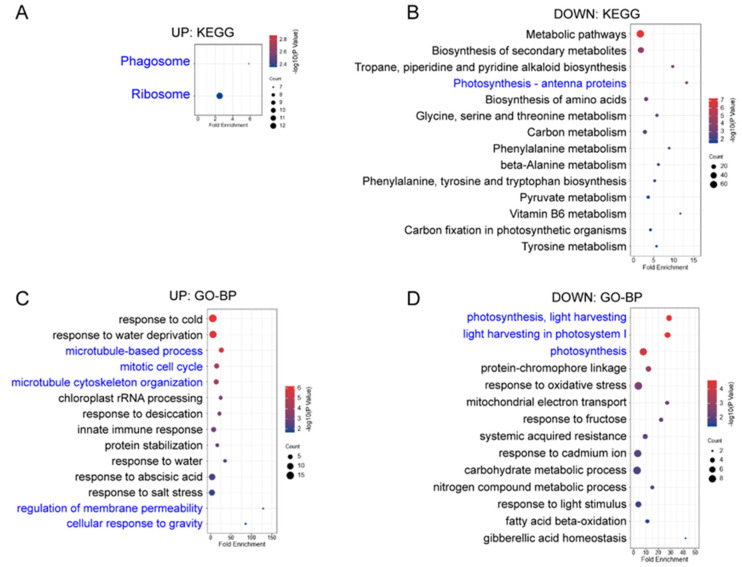
KEGG and GO enrichment analysis of the second repetition of differentially expressed proteins (DEPs-Rep2, ICFI ≥ 1.5) in “*upl5-1* vs. Col-0” group. (**A**,**B**) KEGG enrichment analysis of up-regulated DEPs-Rep2 (**A**) and down-regulated DEPs-Rep2 (**B**); (**C**,**D**) GO enrichment analysis-biological process of up-regulated DEPs-Rep2 (**C**) and down-regulated DEPs-Rep2 (**D**).

The KEGG analysis further showed that the upregulated DEPs were enriched in ribosome and phagosome pathway, and the downregulated DEPs were enriched in photosynthesis antenna proteins and the photosynthesis pathway, as well as calcium signaling (Figure A2). The interesting candidate DEPs were listed in the Figure 5F. For example, the upregulated proteins were most significantly enriched in the JA/calcium signaling/development-associated pathway, such as calcium-dependent protein kinase 32 (CPK32), TSK-associating protein 1 (TSA1), plasma membrane intrinsic protein 2A (PIP2A), plasma membrane intrinsic protein 3 (PIP3), and plasma membrane intrinsic protein 1;5 (PIP1;5), consequently, enriched in the microtubulin pathway such as TUBA4, TUBB4, TUBA3, etc. (Figure 5E,F; Figure A1 and Figure A2); the downregulated proteins were enriched in the photosystem pathway, such as BCA1 (calmodulin-stimulated Ca^2+^-ATPase), PETE1 (Plastocyanin 1), LHCB1.2 (Light harvesting chlorophyll a/b binding protein 1.2), etc. 

When above DUCs (IFCI ≥ 1.5, *p* value < 0.05) were normalized to DEPs (IFCI ≥ 1.5, *p* value < 0.05), total 122 DUCs/DEPs ubiquitination sites (104 proteins) were exhibited (Supplementary Dataset S5). Among them, 103 sites (81 proteins) were downregulated (Figure 5C) from the *upl5*/WT, and only 39 sites (33 proteins) were enriched (Figure 5G). Interestingly, GO analysis of 104 DUCs/DEPs (IFCI ≥ 2, *p* value < 0.05) exhibited UPL5-ubiquitinated proteins were most significantly enriched in the JA/ABA/SA/calcium signaling pathway and the transcriptional regulatory pathway such as H1, H2Axb, BRM, SWI3B, as well as UBP12, PP2A3, etc. (Figure 5H); UPL5-regulated proteins were much less; they enriched in the endomembrane system, such as ACT8, AHA1, HSP70-1, etc. Therefore, it suggests that UPL5 is involved in calcium signaling, the hormone signaling pathway, and the transcriptional regulatory pathway via ubiquitination 26S proteasome pathway to affect plastid and nucleus protein quality or trafficking and control cell senescence.

## 3. Discussion

UPL5 is a typical ubiquitin E3 ligase, UPL5 can ubiquitinate WRKY53 and degrade WRKY53 protein in ubiquitination 26S proteasome pathway and plays a role in leaf senescence and cell death [13,15]. This integrative analysis of proteomic and ubiquitomic datasets reveals UPL5 affects protein quality control of plastid and nuclear proteins and protein trafficking among various components of cells and is involved in the calcium and hormone signaling pathway, determining cell senescence initiation. Although the variant of two replicates was high, we independently analyzed two replicates; 219 DEPs of 130 upregulated and 89 downregulated proteins (|FC| ≥ 1.5) were exhibited in the first replicate; 383 DEPs of 181 upregulated and 182 downregulated proteins (|FC| ≥ 1.5) were shown in the second replicate (Figure A1 and Figure A2). Comparative GO analysis showed same main enrichment category, suggesting the data is sound. 

Except for WRKY53, UPL5 has additional candidate targets in this study, e.g., H2Axb, BRM, SWI3B, CALM (CAM5, CaM4), DSK2, CDC48A, etc. Although we did not exhibit the biochemical evidence in vitro for their ubiquitination, from the facts of declining ubiquitin level of their ubiquitinated sites and increasing protein level of them in *upl5* mutants, as well as subcellular localization of UPL5 in multiple components of the cell, it supposes that they are putative substrates of UPL5 in ubiquitination 26S proteasome pathway. Among them, for H2Axb, BRM, and SWI3B it has been reported that chromatin remodeling ATPases and their associated complexes can alter the accessibility of the genome in the context of chromatin by using energy derived from the hydrolysis of ATP to change the positioning, occupancy, and composition of nucleosomes; they were located in the nucleus [18], which is consistent with the localization of UPL5 in this study (Figure 3 and Figure 5). Although in our previous work UPL5 interacted with WRKY53 in the cytoplasm, perhaps due to cell status under different conditions, it was also found UPL5 regulated *WRKY53* in transcriptional regulation level [13]. Actually, WRKY53 could interact with BRM or SWI3B in pull-down-mass spectrometry analysis assay [19]. Studies have shown that the stability of the BRM protein is regulated by SUMO modifications and proteasomes [20,21], and that SWI3B interacted with deacetylase HD2C to regulate the thermal stress response [22] or participate in transposon silencing with HDA6 in transcriptional regulation level [23], suggesting UPL5 in the nucleus might be involved for chromatin remodeling mediated transcription regulation. 

In this study, UPL5 can also be found in cytoplasm or the nucleus, as well as in plastids in *Arabidopsis* protoplast and tobacco epidermal cell, but until now there is no direct evidence for E3 ligase in plastids. UPL5 modified various patterns of Kub-sites and altered protein level of various components of the cell, that in cytoplasm and plastids can be speculated several possibilities for UPL5 function. Firstly, UPL5 altered CDC48A or CAM5 ubiquitination modification levels to control their functions and the subcellular localization of their downstream proteins. Previous studies have shown that the calmodulin CAM5 is involved in the bicellular localization of two chloroplast proteins, the deletion of the CAM5 gene or the deletion of the essential domain for interaction with CAM5 (CAM domain) alters the subcellular localization of the long-chain fatty acid reductase ceQORH and the chloroplast in vivo membrane protein TIC32. CeQORH is transferred from cytoplasm and nucleus contours to cytoplasm and chloroplasts, while TIC32 changes from cytoplasmic-only localization to cytoplasmic and chloroplast localization [24]. CDC48’s functions involve regulating protein homeostasis, cell cycle regulation, and autophagy, but its primary role is the central driving force behind endoplasmic reticulum degradation [25]. Of the five reported *Arabidopsis CDC48* homologous genes, CDC48A was found to be associated with the chloroplast exoplastid protein TOC33 for ubiquitination and transferring them to cytoplasmic 26S proteasome degradation [26]. Current report showed CDC48 complex can interact with plastid protein RbcL (RuBisCO large subunit) and AtpB (ATP synthase β subunit) and ubiquitinated them under ROS stress condition [27]. At the same time, our proteome dataset showed that the plastid protein and the mitochondrion protein increasingly enriched in *upl5* mutants (Figure 5), thus, UPL5 might alter protein allocation between organelles and cytoplasm. Secondly, UPL5 might be involved in protein trafficking and autophagy pathway in plastid, based on proteome dataset of the *upl5* relative to the WT (Figure A1), when photosynthetic related protein was formed plastid autophagy or vesical to move to vacuole and degrade it. Autophagy and the ubiquitin-proteasome system are the major degradation processes for intracellular components in eukaryotes. Although ubiquitination acts as a signal inducing organelle-targeting autophagy, the interaction between ubiquitination and autophagy in chloroplast turnover has not been addressed, two chloroplast-associated E3 enzymes, SUPPRESSOR OF PPI1 LOCUS1 and PLANT U-BOX4 (PUB4), are not necessary for the induction of either piecemeal autophagy of chloroplast stroma or chlorophagy of whole damaged chloroplasts in *Arabidopsis* [28], however UPL5 acting as a candidate of chloroplast-associated E3 needs to be further addressed. Thirdly, HECT E3s display a wide range of linkage specificities, K6 related to DNA damage and mitochondrion homeostasis, K29 related to epigenetically modification and recognition with UPS, K11 and K48 related to adopt compact conformations, trigger degradation of cell cycle regulators during mitosis, and K63 related protein trafficking leads to endocytosis have been reported in human and bacteria [16]. This study showed that K6, K11, K29, K33, and K63 of UBQ5 were upregulated, and K13, K14, K67, K113, K140, K515, K667, K672, K758, and K662 of CDC48A, as well as K76, K78, K87, and K95 of CAM5 have down/upregulated in *upl5* mutants (Figure 4), supposing that loss of *UPL5* might affect nuclear transcriptional regulation and organelle protein trafficking and degradation. 

Leaf senescence is a programmed dynamic of proteins and metabolites in a variety of cellular processes, including the decline of photosynthetic processes [29,30] and the promotion of calcium/hormone signaling process [31,32,33]. This study has shown the disruption of PETE1 (Plastocyanin 1) and LHCB1.2 (Light harvesting chlorophyll a/b binding protein 1.2) in *upl5* mutants (Figure 5), which affected by chlorophagy or cleaved by proteases [34], leading to the decline of photosynthetic electron transport activity [35,36]. In addition, calcium/hormone responsive proteins including CPK32, TSA1, PIP2A, PIP3, PIP1. 5, etc., which are generally upregulated during leaf aging [37,38,39,40], upregulated in the *upl5*, indicating that UPL5 may be involved in stress-induced leaf senescence via calcium or ABA signaling [13,15].

## 4. Materials and Methods

Wild-type *Arabidopsis thaliana* (L.) Heynold ecotype Columbia, *upl5-1* (SALK_116446), *upl5-2* (SALK_114333), and *UPL5* overexpression line *OE-UPL5-1* and genetic complementation line (*Pupl5:UPL5-1*) plants (kindly provided by Dr. Ulrike Zengtgraf, Tuebingen University, Germany) were grown in greenhouse with a photoperiod of 13 h light/11 h dark, low light (~70–80 μE·m^−2^·s^−1^ at plant height) and an ambient temperature of 21.5 °C. The whole rosette leaves (2 g/part) of 6-week-old plants were collected and frozen in liquid nitrogen, stored at −80 °C for protein extraction. Three independent experiments were completed for collection materials.

### 4.1. Comparative Ubiquitination Modification Proteomics

The standard procedures of comparative ubiquitination-modified proteomics include: preparation of *Arabidopsis thaliana* leaves, extraction of total proteins, trypsin digestion, enrichment of peptides with K-GG antibody, and liquid chromatography-mass spectrometry analysis, data search, and bioinformatics analysis, according to experimental manual of proteomic/ubiquitinomics determination in “Jingjie Bio” company.

The rosette leaves of ten 6-week-old *upl5* and WT plants were pooled and ground in liquid nitrogen into cell powder and then transferred to a 5 mL centrifuge tube, collected in three tubes for one biological replicates, a total of 30 plants were collected for three biological replicates. The specific steps for antibody enrichment with K-GG peptides are as follows: the peptide was dissolved in IP buffer solution (100 mM NaCl, 1 mM EDTA, 50 mM Tris-HCl, 0.5% NP-40, pH 8.0), transferred the supernatant to the pre-washed ubiquitinated resin medium (antibody resin No. PTM1104, from Hangzhou Jingjie Biotechnology Co., Ltd., PTM Bio, China), placed on a rotary shaker at 4 °C, gently shaken, and incubated overnight. After the incubation, the resin was washed four times with IP buffer solution and twice with deionized water. Finally, 0.1% trifluoroacetic acid eluent was used to elute the resin-bound peptide segment, and eluted three times in total. Then, the eluent was collected and vacuum dried. After draining, the salt was removed according to the instructions of C18 ZipTips, vacuum frozen, and drained for LC/MS analysis.

### 4.2. Dataset Collection

MS data were retrieved using MaxQuant (v1.5.2.8) (https://www.maxquant.org (accessed on 2 January 2019). Retrieval parameter settings: the database is UniProt *Arabidopsis thaliana* Columbia ecotype (39,211 sequences), and an opposite-library is added to calculate the FDR caused by random matching, and a common contamination library is added to the database to eliminate contamination in the identification results. The influence of protein; the enzyme digestion method is set to Trypsin/P; the number of missed cleavage sites is set to 4; the error tolerance is 0.02 Da. Cysteine alkylation was set as fixed modification, variable modification as oxidation of methionine, acetylation of protein N-terminus, and ubiquitination of lysine. The FDRs for protein identification and PSM identification were both set to 1%.

### 4.3. Dataset Analysis

Bioinformatic analysis was performed according to previously described protocols [17]. GO term association and enrichment analysis were performed using the Database for Annotation, Visualization, and Integrated Discovery. The KEGG database was used to annotate protein pathways [41]. The KEGG online service tool KAAS was used to annotate the proteins’ KEGG database descriptions. The annotation results were mapped on the KEGG pathway database using the KEGG online service tool KEGG Mapper. The domain annotation was performed with InterProScan on the InterPro domain database via Web-based interfaces and services. WoLF PSORT was used to predict subcellular localization [42]. The CORUM database was used to annotate protein complexes. Motif-X software was used to analyze the models of the sequences with amino acids in specific positions of ubiquityl-21-mers (10 amino acids upstream and downstream of the Kub-site) in all of the protein sequences. In addition, the IPI *Arabidopsis* (*Arabidopsis thaliana*) proteome was used as the background database, and the other parameters were set to default values. The parameter settings for searching motifs using Motif-X software were occurrences 20 and the Bonferroni corrected *p* = 0.005. 

### 4.4. Transient Expression in Arabidopsis Protoplast and Tobacco Leaves

The plasmid *ACTIN:UPL5-GFP* was transformed into the prepared agrobacterium competent cells *GV3101*, respectively, using vacuuming or by injection to infiltrate/inject the *GV3101* bacterial solution carrying the target vector into the prepared *Arabidopsis thaliana* seedlings (4 days old) and tobacco leaves (4 weeks old). The plasmid was isolated using GoldHi Endo Free Plasmid Maxi Kit (CW2104M), then were transformed into the prepared *Arabidopsis* protoplasts by PEG assay, and after 12 h incubation were observed by confocal microscope (Leica SP8, Wetzlar, Germany), the signal parameter GFP (488 nm) was set up. The fluorescence distribution was observed by confocal microscope (Leica SP8) and photographed.

## 5. Conclusions

UPL5 is a typical ubiquitin E3 ligase. UPL5 can ubiquitinate WRKY53 and degrade WRKY53 protein in the ubiquitination 26S proteasome pathway and plays a role in leaf senescence and cell death. This integrative analysis of proteomic and ubiquitomic datasets reveals a landscape of UPL5 mediating ubiquitination: UPL5 affects protein quality control of plastid and nuclear proteins and protein replacement among various components of cells and is involved in calcium and hormone signaling pathway, affecting cell status and cell senescence.

## Data Availability

All datasets have been deposited to the ProteomeXchange Consortium (http://proteomecentral.proteomexchange.org) via the iProX partner repository with the dataset identifier PXD033568.

## Supplementary Materials

The following supporting information can be downloaded at: https://www.mdpi.com/article/10.3390/ijms23105754/s1.
